# Reconstructing the molecular life history of gliomas

**DOI:** 10.1007/s00401-018-1842-y

**Published:** 2018-04-03

**Authors:** Floris P. Barthel, Pieter Wesseling, Roel G. W. Verhaak

**Affiliations:** 1 The Jackson Laboratory for Genomic Medicine, Farmington, CT 06030 USA; 2 Department of Pathology, VU University Medical Center/Brain Tumor Center Amsterdam, Amsterdam, The Netherlands; 3 Department of Pathology, Princess Máxima Center for Pediatric Oncology and University Medical Center Utrecht, Utrecht, The Netherlands

**Keywords:** Glioma, Gliomagenesis, Oncogenesis, Senescence, Telomerase

## Abstract

At the time of their clinical manifestation, the heterogeneous group of adult and pediatric gliomas carries a wide range of diverse somatic genomic alterations, ranging from somatic single-nucleotide variants to structural chromosomal rearrangements. Somatic abnormalities may have functional consequences, such as a decrease, increase or change in mRNA transcripts, and cells pay a penalty for maintaining them. These abnormalities, therefore, must provide cells with a competitive advantage to become engrained into the glioma genome. Here, we propose a model of gliomagenesis consisting of the following five consecutive phases that glioma cells have traversed prior to clinical manifestation: (I) initial growth; (II) oncogene-induced senescence; (III) stressed growth; (IV) replicative senescence/crisis; (V) immortal growth. We have integrated the findings from a large number of studies in biology and (neuro)oncology and relate somatic alterations and other results discussed in these papers to each of these five phases. Understanding the story that each glioma tells at presentation may ultimately facilitate the design of novel, more effective therapeutic approaches.

## Introduction

Gliomas encompass a very diverse group and account for the great majority of tumors originating in the parenchyma of the central nervous system (CNS) [166]. Two larger glioma groups are recognized: so-called diffuse gliomas, characterized by extensive infiltrative growth into the surrounding CNS parenchyma, and more circumscribed (non-diffuse) gliomas such as pilocytic astrocytoma and ependymomas. Diffuse gliomas, by far the most frequent gliomas in adult patients, are traditionally classified according to their microscopic similarities with (precursors of) glial cells and then designated as diffuse astrocytomas, oligodendrogliomas or mixed gliomas/oligoastrocytomas. Additionally, a malignancy grade is assigned to these tumors based on the presence/absence of especially marked mitotic activity, florid microvascular proliferation (MVP), and necrosis [175, 233]. For over a century, such microscopic evaluation has provided the gold standard for the diagnosis of gliomas, assessment of prognosis and formed the basis for therapeutic management. However, multiple studies showed that a purely histopathologic classification suffers from considerable inter- and intraobserver variability [3, 46, 217].

As with other human cancers, the pathogenesis and molecular evolution of gliomas are often characterized by somatic chromosomal aberrations, widespread or focal copy number changes and targeted gain and loss of function events in oncogenes and tumor suppressor genes [211, 224, 225]. Various permutations of somatic alterations were shown to be associated with distinct tumor entities and differential sensitivities to treatment, such as a chromosome 1p/19q-codeletion in oligodendrogliomas conferring increased sensitivity to chemotherapy [26, 27]. In the course of the last two decades, it became increasingly clear that such molecular characteristics may provide a more robust and objective basis for subtyping of diffuse gliomas and both scientists and clinicians turned towards molecular markers to aid diagnosis [30, 34, 59, 234, 244]. Indeed, the International Society for Neuropathology—Haarlem Consensus Guidelines and the most recent edition of the WHO classification of CNS tumors (published in 2016) embraced the notion of an integrated histo-molecular classification of diffuse gliomas [130, 131].

In adult patients three major subgroups of diffuse glioma are now defined based on the presence or absence of mutations in the isocitrate dehydrogenase 1 (*IDH1*) or *IDH2* gene and of complete, combined loss of the short arm of chromosome 1 and of the long arm of chromosome 19 (complete 1p/19q-codeletion):

- IDH-wildtype: most of these histologically represent astrocytic tumors, a large percentage belonging to the highest malignancy grade, i.e., glioblastomas.
- IDH-mutant and 1p/19q-non-codeleted: these tumors also generally have an astrocytic phenotype, but a much larger percentage is at first diagnosis histologically lower grade/WHO grade II or III.
- IDH-mutant and 1p/19-codeleted: most of these are characterized by a prominent oligodendroglial phenotype of the tumor cells.

Following this strategy, the diagnosis of mixed glioma/oligoastrocytoma can be expected to largely disappear, except when additional molecular tests cannot be performed or do not provide unequivocal results; in that situation, not-otherwise-specified (NOS) should be added to the diagnosis to indicate that ideally such samples require further workup [132]. Rare ‘dual genotype’ oligoastrocytomas have been reported that show polymorphic phenotypes with both a complete 1p/19q-codeletion component and non-codeleted component [93, 235]. Diffuse midline glioma, H3 K27M-mutant, was added in the WHO 2016 classification as a separate entity [116, 191, 242]. This type of diffuse glioma, which most often occurs in children, is by definition located in the ‘midline’ of the CNS (brainstem, thalamus, cerebellum and/or spinal cord) and considered as highly aggressive (WHO grade IV) irrespective of the malignancy grade assigned by histology [129]. By far, the most frequent astrocytic tumors in children though are pilocytic astrocytomas that generally are more circumscribed (therefore, grouped under non-diffuse gliomas) and show an indolent, WHO grade I behavior [44, 105, 190]. Meanwhile, the transition from a purely histological to a histo-molecular classification of especially diffuse gliomas represents a paradigm shift and necessitates re-evaluation of histologic criteria used for grading and guidance of therapeutic decisions [164, 181].

**Fig. 1:**
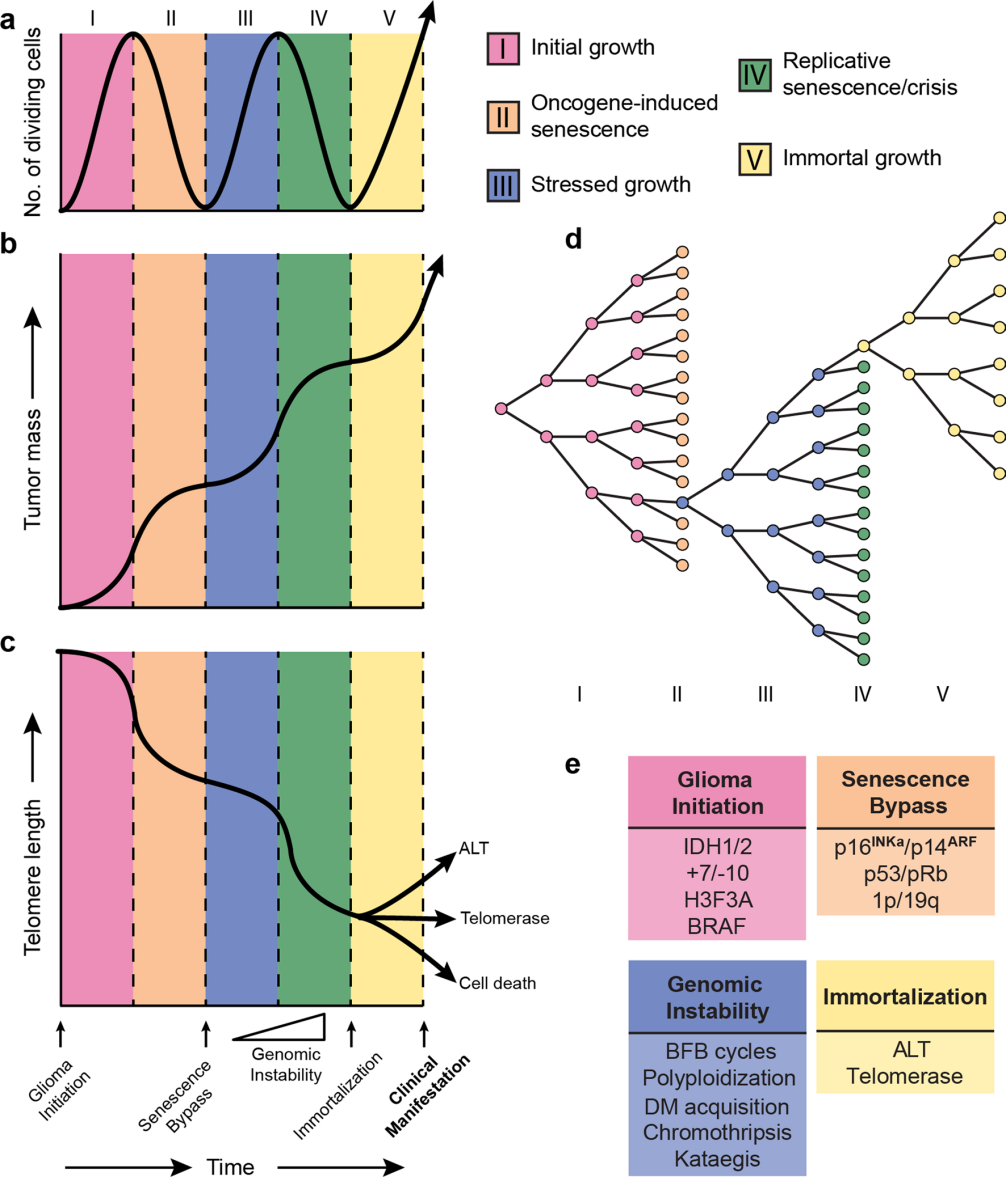
Model of the molecular life history of gliomas, prior to becoming clinically manifest. The temporal sequence of events can be subdivided into five phases (I–V) represented in different colors. **a** The number of dividing cells (or proliferation rate) across each phase. Proliferation peaks towards the end of growth phases and dips going into senescence phases. **b** The tumor mass across each phase. Tumor mass increases exponentially during growth phases and logarithmically during senescence phases. **c** Telomere length across each phase. Telomere length over time follows a pattern that is inverse to tumor mass. **d** Cell doubling diagram indicating the growth barriers (senescence phases) and resulting selection bottleneck. **e** Somatic alterations associated with different phases in gliomagenesis. The timing of each event is indicated on the *x*-axis of panel C. Genomic instability events are accumulated during phase III–IV. Of note, this model is a simplified representation of true gliomagenesis. The *x*-axis is not drawn to scale, in part because the duration of the phases likely varies from cell to cell and between various tumor types. Furthermore, the position of the curves is arbitrary as cells in a tumor may not be in sync. *BFB* breakage–fusion–bridge, *DM* double minute, *ALT* alternative lengthening of telomeres

In this review, we summarize current knowledge and propose five phases in gliomagenesis that occur sequentially and ultimately lead to its clinical manifestation (Fig. 1). Each phase is characterized by distinct molecular alterations and phenotypic characteristics, such as differences in growth dynamics and evolutionary mechanisms. A critical assumption in our model is the existence of two growth barriers, which we refer to as oncogene-induced and replicative senescence. Similar barriers have been described in detail in the context of cultured epithelial cells and fibroblasts and much of this work has paved the road for our understanding of these mechanisms in gliomagenesis [77, 183, 239]. In the model we propose, the first phase of initial growth (phase I) follows the acquisition of a glioma initiation event and is characterized by aberrant proliferation of pre-tumor cells. Continued oncogenic exposure may impede tumor growth and trigger a durable form of cell cycle arrest termed oncogene-induced senescence (phase II) in a majority of tumor cells. Some phase I/II cells may acquire molecular changes to bypass oncogene-induced senescence and continue growth in spite of unfavorable and stressful conditions including DNA damage and dysfunctional telomeres. Continued growth despite incremental genomic instability marks the third phase of stressed growth (phase III). This second round of glioma cell growth under harsh conditions triggers a second round of durable growth arrest termed replicative senescence (phase IV) and in some cases, brings forth a state of cellular crisis characterized by widespread cell death. Rare cells may acquire stem-like characteristics and a means to continue growth indefinitely, giving rise to a final phase of immortal growth (phase V). Such phase V glioma stem-like cells uphold the tumor progenitor cell population via their capacity for self-renewal and may also give rise to more differentiated and growth-arrested stage IV cells, losing their stem-like properties. In this manuscript, we systematically review the evidence for this model and propose candidate mechanisms where definitive evidence is lacking. Acknowledging that this model is an abstract simplification of gliomagenesis, we also provide some examples of exceptions and conflicting evidence. While the focus of this review is on various molecular categories of diffuse glioma recognized by the most recent WHO classification, a few examples in the realm of non-diffuse gliomas are touched upon as well.

## A model for the temporal molecular pathogenesis of gliomas

### Phase I: initial growth

The theory that cancer results from accumulation of mutations over time, in a subset of patients combined with contribution of inherited risk factors, has been around for over six decades and has been refined over the years [5, 79, 80, 111, 158, 160]. For the purpose of this review we will consider a glioma initiation event to be the first acquired (somatic) event towards developing glioma. This event should provide a competitive growth advantage, either by directly increasing proliferation or by creating the conditions in which increased proliferation may happen. Cells and their progeny characterized by such an event will be primed to outcompete neighboring cells giving rise to an initial submicroscopic tumor mass. While germline events that contribute to glioma risk precede such glioma initiation events, their incomplete penetrance suggests that they cannot be considered as causal for glioma formation and instead prime the environment for tumor formation [196]. Furthermore, even if a glioma initiation event marks the first somatic event in the formation of a tumor it may not be responsible for initiating growth directly. Instead, this event may promote tumorigenesis indirectly via stochastic activation of oncogenes or repression of tumor suppressors. Genetic or epigenetic selection pressures will prioritize daughter cells with growth advantages over those without and daughter cells with lethal genotypes will rapidly disappear [64, 150, 245].

#### IDH-mutant diffuse gliomas

Mutations in *IDH1*/*IDH2* are commonly considered to be glioma initiating. Several studies have shown that they are amongst the few alterations highly shared amongst gliomas at first presentation and their recurrences [9, 100, 204] which is explained by their presence in the cell of origin and all cells derived thereof. Comparing multiple biopsies from the same tumor, IDH mutation can be confidently detected in each tumor segment and thus fit the proposed criteria of a glioma initiation event [107, 124, 204]. In vitro experiments have demonstrated that IDH mutations alone are sufficient to reprogram the transcriptome and epigenome of normal cells to prevent these cells from entering a terminally differentiated state [134, 147, 213].

IDH dysregulation likely contributes to gliomagenesis via the accumulation of the oncogenic metabolite R(−)-2-hydroxyglutarate (2HG) [114, 176]. Wildtype IDH enzymatically converts isocitrate into α-ketoglutarate (α-KG) as part of the citric acid cycle, whereas mutant IDH metabolizes α-KG into 2HG [54, 231]. Both mutant and wildtype IDH alleles are, therefore, essential for the oncogenic function of IDH. IDH mutations in glioma result in genome-wide hypermethylation [159, 213], most likely due to effects of 2HG on the ten–eleven translocation methylcytosine dioxygenase (Tet) family of proteins [61, 134, 243]. This hypermethylation may provide a growth advantage to cancer cells due to the epigenetic activation of oncogenes via stochastic activation of alternative gene regulatory programs, some conferring added fitness [64]. One such mechanism in glioma may be through methylation-induced disruption of a CCCTC-binding factor (*CTCF*) binding site, resulting in aberrant activation of platelet-derived growth factor receptor alpha (*PDGRFA*) [63].

Although most experimental models of IDH involve overexpression of mutant IDH in vitro, several transgenic mouse models have been described [28, 125]. Early transgenic models showed that conditional knock-in of mutant IDH in the murine brain led to perinatal lethality [188]. A more recent inducible model demonstrated that mutant IDH led to increased proliferation and infiltration in the CNS parenchyma of murine neural stem cells [10]. Though these mice eventually died due to hydrocephalus and did not develop malignant tumors, they showed symptoms of the initial phases of gliomagenesis. Thus, while experimental models of mutant IDH are generally insufficient to cause glioma, mutant IDH leads to changes that could be interpreted as early tumor development.

#### IDH-wildtype diffuse astrocytomas

Approximately 70% of IDH-wildtype diffuse astrocytomas are characterized at the molecular level by a single copy loss of chromosome 10 and gain of chromosome 7 (+ 7/− 10) [16, 34]. Loss or diploid loss of heterozygosity of chromosome 10 and of chromosome arm 10p in particular has been reported to occur more frequently and may precede gain of chromosome 7 [94]. Based on evolutionary modeling using primary-recurrent tumor pairs and multisector tumor sampling, several independent groups have found that + 7/− 10 is homogeneous and longitudinally preserved and thus likely the first and glioma initiation event in a large fraction of IDH-wildtype diffuse astrocytomas/glioblastomas [69, 107, 168, 198, 229]. A recent study suggested that gains of chromosome 7 likely occur early in tumorigenesis, amongst the first 10% of driver events [69]. In the past several years, there has been a lot of interest in the role of *TERT* promoter mutations in oncogenesis and an increasing body of evidence suggests that these mutations precede + 7/− 10 [106]. Nevertheless, a potential role of *TERT* promoter mutations to promote proliferation in the initial growth phase and as a glioma initiation event is speculative and will be discussed later in this review.

Chromosome 7 is home to several oncogenes that have been implicated in gliomagenesis such as cyclin-dependent kinase 6 (*CDK6*), MET proto-oncogene (*MET*) and epidermal growth factor receptor (*EGFR*), while chromosome 10 hosts several tumor suppressor genes, including Tet family member Tet methylcytosine dioxygenase 1 (*TET1*) and phosphatase and tensin homolog *(PTEN)*. Though these genes comprise the prime suspects, it is unlikely that they alone are responsible for initiating glioma development [168]. Studies across different diseases and in various model organisms have shown that large chromosomal copy number changes led to gross gene dosage fluctuations impacting various specific and general cellular functions [206]. Such changes may, therefore, act in concert to promote tumor development.

Some IDH-wildtype diffuse gliomas show cytogenetically intact chromosomes 7 and 10, implying that other initiation events give rise to these tumors as well. Such events may include activating or inactivating alterations in the phosphoinositide 3-kinase (PI3K), receptor tyrosine kinase (RTK) and mitogen-activated protein kinase (MAPK) pathways [142]. PI3K pathway alterations include mutations in PI3-kinase subunit alpha (*PIK3CA*), PI3-kinase subunit P85-alpha (*PIK3R1*), or inactivation the aforementioned tumor suppressor *PTEN* [127, 173, 199]. Glioma initiation events may also include point mutations in RTK pathway genes such as *EGFR* and *PDGFRA* or in MAPK pathway genes such as neurofibromin 1 (*NF1*) [220]. Much is already known about the effect of these mutations on cancer growth but additional research is needed to secure their potential role as glioma initiation events.

A particular subgroup of diffuse IDH-wildtype gliomas is characterized by mutations in H3 histone family members and these gliomas occur most often in children [191, 202, 241]. The diffuse midline glioma, H3 K27M-mutant, shows a lysine to methionine substitution at position 27 of the H3 histone family member 3A (*H3F3A*) or histone cluster 1 H3 family member 3B (*HIST1H3B*) gene and is included in the WHO 2016 classification as a separate entity. Other H3-mutant diffuse gliomas in children and adolescents occurring predominantly in the cerebral hemispheres often show H3 G34R/V mutation (implying a glycine 34 to arginine or valine substitution) [115, 201, 202, 241]. In contrast to hypermethylated IDH-mutant gliomas, these H3-mutant gliomas have a general DNA hypomethylation phenotype [14]. A recent study showed that expression of mutant H3 K27M in neonatal mice brains led to ectopic proliferation, indicating a possible pre-cancerous change [126]. Although additional support is needed, combined with their apparent mutual exclusivity with mutations in IDH and changes characteristic for IDH-wildtype tumors, these findings indicate that H3 K27M and H3 G34R/V mutations may be glioma initiation events [32].

#### Non-diffuse gliomas

Recent studies have shown that pilocytic astrocytomas near universally harbor abnormalities in the MAPK pathway, and most commonly a tandem duplication targeting chromosome 7q, which gives rise to a *KIAA1549*–*BRAF* fusion gene consisting of the N terminus of *KIAA1549* and the kinase domain of v-RAF murine sarcoma viral oncogene homolog B1 (*BRAF*) [44]. Alternative alterations include the oncogenic V600E missense mutation also targeting *BRAF* [190]. The *BRAF* V600E mutation results in an activating change due to a substitution of valine with glutamic acid at codon 600. In a non-cancer setting, *BRAF* activates kinases MEK and ERK, which in turn activate transcriptional machinery to promote differentiation, proliferation, growth and apoptosis [31]. Both *BRAF* V600E mutation and *BRAF* fusion genes contribute to tumorigenesis by constitutively activating the kinase domain of *BRAF*, resulting in overactive signaling activity and a selective growth advantage for affected cells [55, 67, 102]. In most pilocytic astrocytomas (even after thorough analysis), an activating change in *BRAF* or other MAPK pathway members is the only genomic change that can be confidently detected, implying that it is the glioma initiation event in this disease [187].

### Phase II: oncogene-induced senescence

**Fig. 2:**
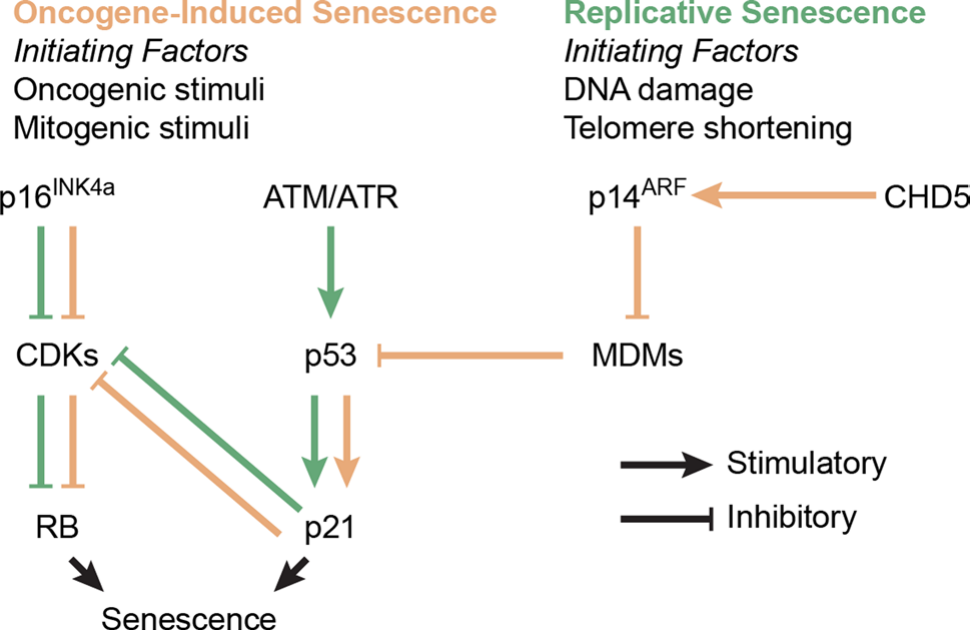
Process diagram indicating the p16^INK4a^/p14^ARF^–RB–p53 pathway in normal conditions. Disruption of one or multiple components through mutation or copy number change may prevent or suppress the onset of senescence. Various stimuli use different routes to activate the senescence response, leaving compensatory mechanisms in place in case components fail. For example, if oncogene-induced senescence is repressed via *CDKN2A/B* inactivation, DNA damage and telomere shortening could still trigger replicative senescence via *ATM* and *ATR*. *CDKs* cyclin-dependent kinases (e.g., CDK2), *MDMs* murine double minutes (e.g., MDM2)

Continued oncogenic signaling in the initial growth phase prompts the activation of tumor suppressive signaling via activation of the p16^INK4a^/p14^ARF^–RB–p53 cell cycle and cell stress pathways (Fig. 2), slowing tumor growth and transitioning a majority of cells with intact pathways into a terminal state called oncogene-induced senescence [43]. First discovered over five decades ago in cultured fibroblasts, senescence is a stress-induced durable cell cycle arrest [82]. The role of senescence in cancer has been reviewed extensively [29, 42, 78, 83, 174, 193]. Briefly, senescence provides a major tumor suppressive barrier and dividing tumor cells are put under selection pressure to acquire molecular events to prevent or overcome its onset. Hallmarks of senescence include durable growth arrest; short, dysfunctional telomeres; and a marked increase in DNA damage and stress signaling [42]. Although senescent cells are growth arrested, they are metabolically active and release a plethora of signaling molecules to the microenvironment, also known as the senescence-associated secretory phenotype [174]. Distinction must be made between oncogene-induced senescence which is discussed here and is triggered by chronic oncogenic signaling, and replicative senescence discussed later, describing senescence triggered by telomere dysfunction following extensive replicative cycles [193]. Oncogene-induced senescence poses a significant growth barrier and most cells will not acquire molecular alterations that allow them to bypass this barrier and will, therefore, become senescent [43]. However, rare cells may acquire such alterations, eliciting a selective sweep by a subclone that will rapidly dominate the neoplastic cell population.

In some cancer types, a senescent precursor stadium can be identified, such as intestinal polyps in colon cancer and dysplastic naevi in melanoma [192]. In contrast to the catastrophic karyotypes demonstrated by their later stage derivatives, these growth-arrested senescent precursor lesions entered a senescent state via the activation of single oncogene such as *BRAF* and are otherwise genetically unremarkable [72, 117, 151, 153]. This begs the question if such premalignant precursor changes exist for diffuse gliomas as well. If so, such precursor lesions may, however, well be (sub)microscopic in size and go unnoticed in imaging or autopsy studies.

The tumor suppressor proteins p16^INK4a^, p14^ARF^, RB and p53 can be considered as gatekeepers of senescence. The INK4a/ARF locus on chromosome 9 contains both cyclin dependent kinase inhibitor 2A (*CDKN2A*) and 2B (*CDKN2B*), combined encoding for both p16^INK4a^ and p14^ARF^ via alternative splicing. Tumor suppressors RB and p53 on the other hand are encoded for by the genes retinoblastoma 1 (*RB1*) and tumor protein 53 (*TP53*) on chromosomes 13 and 17, respectively. Inactivation of one or multiple of these genes via genomic deletion and/or inactivating mutations has been linked to repression of senescence signaling and is a common event in all cancers including gliomas [39, 83, 195, 240]. For the purpose of this review, we define the term ‘senescence bypass event’ as any molecular alteration that suppresses the onset of oncogene-induced senescence.

#### IDH-mutant diffuse astrocytomas

IDH-mutant astrocytomas are often characterized by loss of one allele of *TP53*, combined with a loss-of-function mutation in the remaining allele. The frequency of *TP53* mutations in IDH-mutant (‘secondary’) glioblastomas is comparable to that in lower grade IDH-mutant astrocytomas from which these glioblastomas are derived via malignant progression, suggesting that *TP53* aberrations are early lesions in these tumors [162]. Furthermore, and in contrast to IDH-wildtype gliomas, *TP53* mutations were shared between all *TP53*-mutant cases of primary and recurrent tumors in a recent study [100]. Analysis of multiple biopsies from the same IDH-mutant tumors indicated that samples mutant for *TP53* were always IDH mutant, while some IDH-mutant samples lacked *TP53* mutations, suggesting that IDH mutations precede *TP53* inactivation [232]. IDH mutation and *TP53* inactivation, therefore, both comprise early events in gliomagenesis, with *TP53* inactivation generally following mutation in IDH.

The p53 tumor suppressor protein is involved in many different functions, and especially its role in cell cycle arrest and senescence is very well understood [81]. While enzymatically active wildtype p53 triggers senescence in response to oncogenic stress, mutant p53 inadequately blocks proliferation thereby bypassing the onset of oncogene-induced senescence. In addition, p53 takes a prominent role in the senescence pathway (Fig. 2) and loss of its enzymatic activity impacts replicative senescence, triggering crisis [195]. This is discussed further in phase IV (“Phase IV: Replicative senescence/crisis*”* section).

#### IDH-mutant oligodendrogliomas, 1p/19q-codeleted

The majority of IDH-mutant tumors wildtype for *TP53* demonstrate a combined single copy loss of the complete chromosome arms 1p and 19q (complete 1p/19q-codeletion) [20, 27]. These tumors are the canonical oligodendrogliomas according to the revised WHO criteria [130]. Though it has been suggested that 1p/19q-codeletions are the result of an unbalanced translocation, much about the contribution of this event to oncogenesis remains to be resolved [95]. 1p/19q-codeletion was found to be stable across longitudinal samples and multiple biopsies, suggesting that they are early events [1, 97, 218]. The finding that codeleted tumors are almost exclusively IDH-mutant, while the reverse is not true, suggests that mutations in IDH precede codeletion. This suggestion may implicate a common cell of origin for both astrocytoma (IDH-mutant, non-codeleted) and oligodendroglioma (IDH-mutant and 1p/19q-codeleted). Indeed, recent evidence was provided that substantiates this hypothesis, demonstrating that differences in inferred cell identity between oligodendrogliomas and astrocytomas may be entirely explained by different microenvironment makeup and the impact of the 1p/19q-codeletion on the expression of genes on these chromosome arms [219]. The authors found that although these histological tumor types differ in morphology, these histological subtypes share a comparable developmental hierarchy and glial lineage. Transcription factors involved in oligodendrocyte differentiation are also expressed in both histologies [6]. Several case reports have highlighted ‘dual genotype’ oligoastrocytomas, demonstrating molecular features of both bona fide astrocytic and bona fide oligodendroglial tumor cells [93, 235]. Such a dual genotype may be explained by assuming that very early in gliomagenesis a subset of IDH-mutant cells experiences a complete 1p/19q-codeletion and a *TERT* promoter mutation, while another IDH-mutant subset acquires a mutation in *TP53* and/or *ATRX*. Detailed and sufficiently powered longitudinal studies of primary and recurrent diffuse gliomas may help to elucidate the actual frequency of such ‘dual genotypes’, how they are related to mixed histological appearances, and how they evolve over time [2].

Additional loss-of-function mutations in far-upstream element binding protein (*FUBP1*) on 1p31.1 and capicua transcriptional repressor (*CIC*) on 19q13.2 are observed in over 60% of 1p/19q-codeleted gliomas [15]. A paired analysis of primary and recurrent 1p/19q-codeleted oligodendrogliomas described distinct alterations in *CIC* and *FUBP1* in the primary and the recurrent tumor [9]. Another report described that these events were frequently private to either primary or recurrence, but not both [1]. Both findings corroborate observations of *CIC* and *FUBP1* mutation heterogeneity across nine distinct samples from the same oligodendroglioma, including finding five distinct *CIC* mutants across nine tumor samples [204]. Loss-of-function mutations in these genes led to a loss of protein expression and the *FUBP1* mutation was associated with adverse survival compared to wildtype tumors [35, 90]. These findings suggest an important role for these genes and indicate that in cases in which these events were not found in one sample of the tumor, they still might be present elsewhere. Despite their apparent importance, the role of these events in gliomagenesis remains to be understood.

It is not clear whether 1p/19q-codeleted oligodendrogliomas undergo senescence or acquire mechanisms to bypass senescence. 1p/19q-codeleted gliomas generally lack alterations in genes associated with oncogene-induced senescence such as *BRAF* or senescence bypass such as *TP53*, *RB1* or *CDKN2A*. Nevertheless, given their continued clinical growth it appears that these tumors have somehow evaded growth arrest and senescence barriers. Perhaps the 1p/19q-codeletion allows pre-cancerous cells to avoid senescence through the mono-allelic inactivation of tumor suppressor genes on these chromosome arms [197]. One mechanism that was previously proposed may be via a dosage-dependent repression of chromodomain helicase DNA-binding domain 5 (*CHD5*) on 1p36 [7, 8, 222]. A study that used genetic engineering to create mouse models with gains and losses of a region corresponding to human 1p36 found that duplication of this region led to decreased proliferation and senescence whereas a single-copy deletion led to immortalization [8].

#### IDH-wildtype diffuse astrocytomas

Amongst IDH-wildtype astrocytomas/glioblastomas, one of the most frequent alterations is a homozygous loss of *CDKN2A* and *CDKN2B* [128]. Mathematical modeling has suggested that homozygous *CDKN2A/B* loss occurs after +7/−10 but before other molecular events [168]. Homozygous *CDKN2A/B* loss alone is insufficient for tumor formation in mice, requiring the activation of an oncogene to generate tumors in vivo [215]. As such, homozygous *CDKN2A/B* loss is likely a second event in the tumorigenesis of IDH-wildtype astrocytoma or glioblastoma. The role of protein products p16^INK4a^ and p14^ARF^ in senescence is very well understood. Indeed, astrocytes with a homozygous deletion of *CDKN2A/B* can grow indefinitely in culture, and introduction of p16^INK4a^ in immortal human glioma cell lines with this deletion leads to cell cycle arrest and senescence [87, 216]. Taken together, these results indicate that loss of *CDKN2A/B* may provide adult IDH-wildtype astrocytomas with a reliable means for senescence bypass.

Mutations in *TP53* sometimes co-occur with homozygous *CDKN2A/B* loss in IDH-wildtype glioma [163]. While *TP53* mutations are often shared across all tumor cells in IDH-mutant astrocytoma, *TP53* mutations in IDH-wildtype astrocytomas are frequently unique to one or a few tumor subclones [107]. In this same study, it was found that amongst IDH-wildtype astrocytomas, whereas *CDKN2A/B* is consistently deleted, *TP53* mutations are frequently lost or gained at tumor recurrence. These observations suggest that, in IDH-wildtype astrocytoma, *CDKN2A/B* may be primarily important for senescence regulation

Pediatric H3-mutant/IDH-wildtype diffuse gliomas are *TP53* mutant in about 50% of cases, which may act as a senescence bypass event in these tumors [191]. Amongst remaining H3-mutant tumors, approximately 20–30% of H3 K27 mutant diffuse gliomas show mutations in Activin A Receptor Type 1 (*ACVR1*) [23, 65, 208, 242]. There is no evidence that *ACVR1* has any role in sentence regulation; however, *ACVR1* alterations were found to be mostly mutually exclusive with alterations in *TP53* and *PPM1D*. *PPM1D* is a protein phosphatase downstream of p53 involved in apoptosis regulation following DNA damage and thus likely involved in senescence [62]. Expression of *ACVR1* mutants in Tp53^null^ murine astrocytes implanted in mouse brains failed to induce tumors, likely because H3 K27 mutations are required as a tumor initiation event and thus suggesting that *ACVR1* does not meet the criteria for a tumor initiation event [242]. Thus, while about half of pediatric H3-mutant gliomas demonstrate *TP53* mutations that may act to bypass senescence, it remains unclear if and how *TP53*-wildtype H3-mutant pediatric gliomas bypass senescence.

#### Non-diffuse gliomas

Several lines of evidence suggest that pilocytic astrocytomas (WHO grade I, IDH-wildtype) are arrested in a senescent phase II state and do not advance to later phases. First, these tumors were found to frequently demonstrate several biomarkers of senescence at tumor detection, including widespread β-galactosidase activity and p16^INK4a^ staining [88, 112, 177]. Second, these tumors are very quiescent genetically, often demonstrating but a single activated oncogene, such as a *BRAF* fusion, *BRAF* V600E mutation or rarely an activating mutation in *FGFR1* or *PTPN11* [101, 212, 246]. Third, these tumors generally grow slowly, have excellent outcomes and sometimes regress, perhaps because the tumor cells do not immortalize [24, 25, 75, 185]. Fourth, expression of activated *BRAF* V600E alone does not lead to tumor development in in vitro and in in vivo mouse models, while the combined activation of *BRAF* and loss of *CDKN2A/B* is transforming, suggesting that additional mutations to bypass senescence are required to advance to phase III [92, 182, 189]. The clinical presentation of pilocytic astrocytoma may be the result of senescence-mitigating circumstances, such as a cell of origin with proliferative potential in the absence of senescence-bypass.

### Phase III: stressed growth

Cells presenting with continued proliferative signaling beyond the oncogene-induced senescence barrier are generally characterized by defective DNA damage response signaling and continued growth in a stressed environment. During this phase the repetitive DNA at the telomeric terminal ends of chromosomes become increasingly important [161]. Telomeres progressively shorten as cells divide due to the linear conformation of chromosomes and directional replication machinery, a phenomenon that is critically important for diseases like cancer which are characterized by often rampant proliferation [165]. Telomeric DNA takes on a lasso conformation called the t-loop, and these loops are bound by the shelterin DNA-binding protein complex. Together, these characteristics protect chromosome ends from being recognized as DNA double-strand breaks and prevent inadvertent activation of DNA damage response pathways [56].

**Fig. 3:**
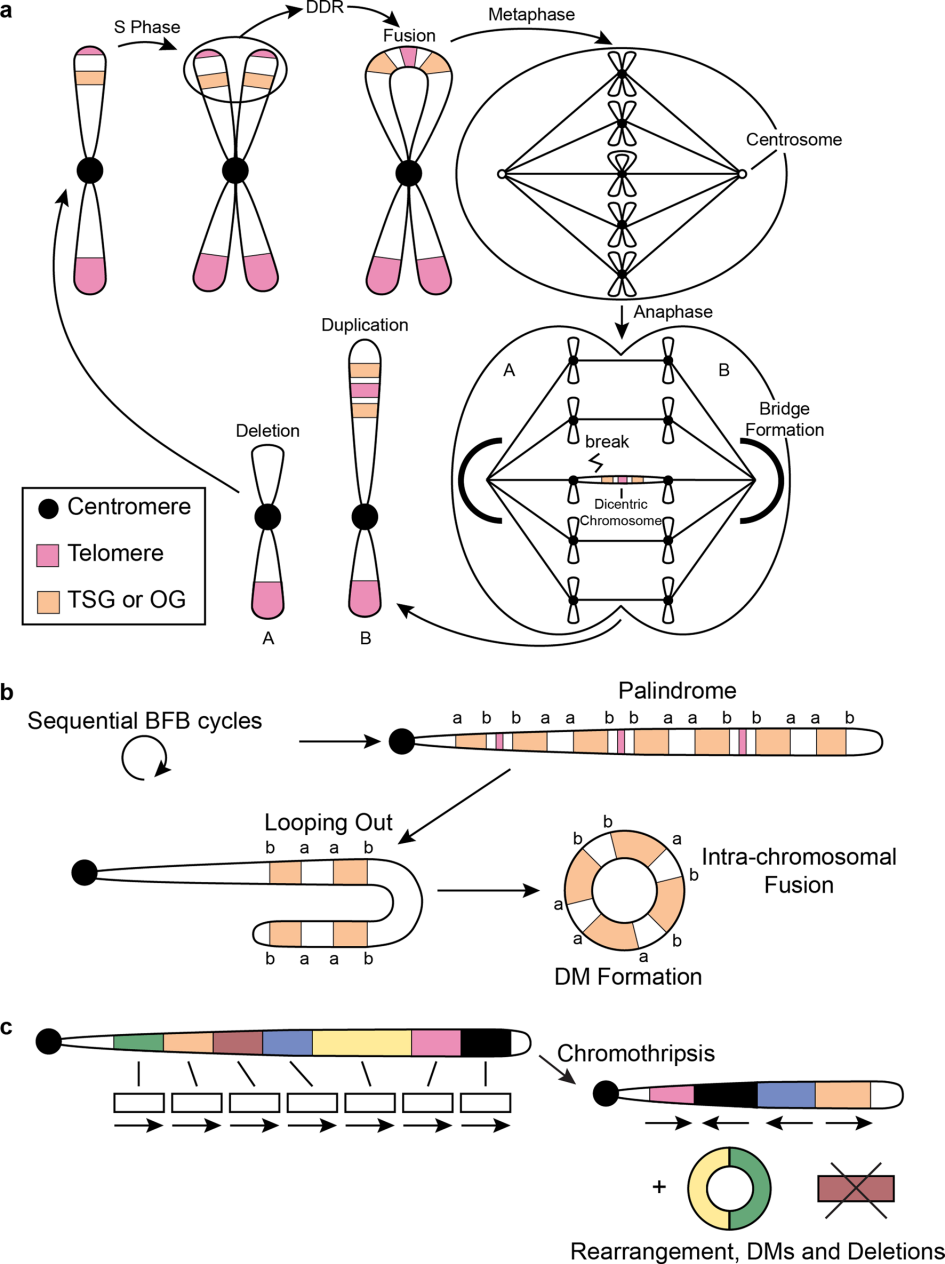
Genomic instability related to telomere stress. **a** Schematic illustrating BFB cycles. Following a single BFB cycle, daughter cells are left with unequal DNA content, leading to a deletion in A and an amplification in B. BFB cycles may also involve fusion of non-sister telomeres (not shown). **b** Repetitive BFB cycles form palindromes demonstrating high intra-segmental homology. This can lead to intra-chromosomal fusions and formation of double minutes. **c** Following chromothripsis segments can be rearranged, lost or circularized. *TSG* tumor suppressor gene, *OG* oncogene, *DDR* DNA damage response, *DM* double minutes, *BFB* breakage–fusion–bridge

Dysfunctional telomeres are critically short and improperly protected telomeres lacking t-loops and shelterin complexes. They trigger the activation of DNA damage response pathways via ataxia telangiectasia-mutated (*ATM*) and ataxia telangiectasia and Rad3-related (*ATR*) kinase, which are triggered by exposed and unprotected double-stranded and single-stranded DNA break ends, respectively. The exposed ends then fall victim to homology directed repair (HDR) and non-homologous end joining (NHEJ) repair processes, intended to repair accidental DNA breaks but lead to gross genomic instability when triggered by dysfunctional telomeres. When telomeres are unprotected, these repair processes prompt sister chromatids to fuse with one another, forming a dicentric chromosome. During the anaphase, the dicentric chromosome will form a bridge spanning the mitotic spindle and connecting the two daughter cells. Resolution of the chromatin bridge via cytoplasmic 3′ nuclease *TREX1* results in breakage of the dicentric chromosome at a locus not necessarily at the site where the fusion had occurred, resulting in an unbalanced inheritance of genetic material between the two daughter cells. Because the resulting daughter cells also lack telomeres, this process of breakage–fusion–bridge (BFB) cycles (Fig. 3a) will repeat itself every subsequent cell division until telomeres are restored [4, 70, 144, 148, 149]. The detrimental genomic instability acquired via telomere dysfunction and BFB cycles endows these cells with powerful stochastic mutational mechanisms to acquire changes that provide a survival benefit under selective pressure.

The intensity of genomic instability endured during the stressed growth phase may depend on the severity of senescence pathway dysregulation incurred overcoming oncogene-induced senescence (Fig. 2). In the case of H3-mutant, IDH-wildtype and IDH-mutant astrocytoma/glioblastoma this pathway is perturbed close to the source via the direct loss of RB, p53 or p16^INK4a^ protein function. In IDH-mutant and 1p/19q-codeleted oligodendroglioma this pathway may be repressed indirectly, for example, via the modulation of p14^ARF^ activity through a partial deletion of *CHD5*. This may explain why the latter group of tumors shows significantly less genomic instability compared to the former tumor types. Moreover, in pilocytic astrocytoma this pathway may not be affected at all and these tumors may not advance beyond oncogene-induced senescence. While genomic instability incurred during phase III demonstrates some patterns that are unique to a certain glioma subtype, these features are generally shared across all gliomas regardless of subtype. We, therefore, did not separate this section according to tumor type as we did for the other phases. More research is needed to carefully delineate the selective pressures at play to better understand differences and similarities between various glioma entities in this respect.

The advent of high-throughput sequencing has led to remarkable progress in understanding the complexity of genomic instability in cancer, including complex deletions, amplifications and translocations [245]. It is important to note that the genomic organization that can be reconstructed using sequencing at the time of analysis are those changes that resulted in viable cells and were selected for. Recent work has shown that telomere dysfunction directly leads to catastrophic genomic events, including genome shattering (chromothripsis), clustered regions of focal hypermutation (kataegis) and whole genome doubling (tetraploidization) [60, 137, 138]. Although the incidence of chromothripsis across the spectrum of gliomas is not known, a recent report suggests that chromothripsis is very common in glioblastoma [139]. Comparison of primary and recurrent tumors across various tumor types including gliomas demonstrated that recurrent tumors lack additional genomic instability, suggesting that these events occurred during a stressful period, the initial development of the tumor that was later stabilized [60].

Recently, there has been a renewed interest in circular extrachromosomal DNA elements called double minute (DM) chromosomes in cancer, and it was shown that such DMs are common in gliomas [57, 214]. Although DM chromosomes have been long recognized as a cytogenetic feature of cancer, relatively little was known about its biological relevance. DM chromosomes have a predisposition to involve cancer oncogenes such as MYC proto-oncogene protein (*MYC)*, MDM2 proto-oncogene (*MDM2*) or cyclin-dependent kinase 4 (*CDK4)* [53, 113, 156, 186, 247]. A unique feature of DM is that they lack centromeres and telomeres to dictate the organization of the mitotic spindle during mitosis and are, therefore, randomly distributed across daughter cells [103]. Interestingly, this feature hypothetically provides DM chromosomes with an impressive fitness advantage over linear chromosomes as they do not need telomeres to protect them from inadvertent DNA damage response pathways and are not subjected to detrimental BFB cycles. It has been proposed that DM chromosomes are the result of the fusion and circular assembly of stretches of linear DNA consisting of highly homologous sequences of inverted duplications in tandem following sequential BFB cycles (Fig. 3b) [205]. Others have proposed that ineffective DNA repair following chromothripsis can lead to linear DNA fragments getting pieced together in a circular fashion (Fig. 3c) [66]. A study by our group found evidence of chromothripsis in several glioblastoma samples localized to chromosome 12 involving *MDM2* and *CDK4* and suggested that these segments may be arranged in extrachromosomal DMs [247]. Because these are inherently random processes, it is possible that DM chromosomes that promote survival are positively selected for during telomere dysfunction. Compared to IDH-mutant gliomas, DMs in IDH-wildtype tumors more often involve established glioma oncogenes, despite what appears to be a comparable frequency of DMs in both glioma categories [57]. More research is needed to precisely determine the frequency of DMs in glioma subtypes and to pinpoint the genetic origin of these structures.

A recent study of paired primary and recurrent IDH-mutant gliomas reported that in some cases allelic imbalances of the IDH-mutant allele occurred upon recurrence, which led to a change in mutant protein expression and consequently decreased 2HG production [145]. Furthermore, IDH-mutant tumors are very hard to culture, and when it succeeds, IDH mutations that were present initially have been reported missing, raising the possibility that losing an IDH mutation is advantageous for survival in culture [135]. Introduction of mutant IDH in cell cycle checkpoint-deficient cells rapidly transforms these cells into competent tumor cells [99]. However, IDH inhibition in these cells after as little as 4 days after its first introduction did little to slow tumor growth. These findings suggest that some IDH-mutant gliomas may rapidly evolve and acquire additional driver events to uphold the tumor cell population.

To summarize, telomere dysfunction and stressed growth may promote the context-dependent evolution of glioma cells, sometimes rendering glioma initiation events redundant and providing gliomas with new fuel that rapidly increase intratumoral heterogeneity and can deal with various toxic stresses and bottlenecks. While stochastic mutational mechanisms in the stressed growth phase provide ample selection pressure to acquire beneficial changes, the detrimental genomic instability under which cells must operate acts as a powerful tumor suppressive barrier. Unchecked growth will rapidly lead to another round of DNA damage-induced replicative senescence, or when those checkpoints fail completely, cell crisis.

### Phase IV: replicative senescence/crisis

Sustained stressed growth is not durable and will eventually lead tumor cells down to one of two possible roads. Tumor cells with a partially intact senescence response (i.e., functional p53 and RB) may undergo a second round of senescence called replicative senescence in response to dysfunctional telomeres. Tumor cells with a completely dysfunctional senescence response (i.e., loss-of-function mutation in *TP53* or *RB1*) instead continue proliferating in a state of cellular crisis leading to cell death in a vast majority of cells [51]. Replicative senescence and crisis both pose a second population bottleneck to further tumor formation. It is essential that tumor cells transition to a less stressful environment with proper telomere maintenance to prevent further BFB cycles and other catastrophic events. Cell culture experiments have demonstrated that direct immortalization of cells prior to a stressed growth phase enables them to bypass genomic instability and immortalize lacking the wild karyotypes typically associated with malignant transformation [68, 154]. These observations suggest that genomic instability in cancer development generally precedes immortal growth and is required to generate errors enabling telomere maintenance [68].

Acquisition of a telomere maintenance mechanism endows cancer cells with immortal growth, meaning that they are bestowed a limitless replicative potential [108]. Telomere maintenance is established once a tumor cell has reactivated telomerase or activated alternative lengthening of telomeres. Moreover, restoration of telomere function may prevent further BFB cycles and restore genome stability. The canonical pathway involves the reactivation of the ribonucleoprotein telomerase which is transcriptionally silent in differentiated adult cells [74]. The telomerase catalytic component telomerase reverse transcriptase (*TERT*) is expressed in over 80% of human cancers and is thought to be rate limiting for telomerase activity [194]. In the alternative pathway tumors become immortalized via a recombination-driven mechanism called alternative lengthening of telomeres (ALT) [21].

#### IDH-mutant diffuse astrocytomas

IDH-mutant diffuse astrocytomas almost universally demonstrate ALT [85]. ALT cells present with several defining characteristics, including a heterogeneous distribution of telomere length across chromosomes, extrachromosomal telomeric DNA fragments in a circular configuration (c-circles), increased expression of telomeric repeat-containing RNA (*TERRA*) from telomeres, the formation of ALT-associated promyelocytic leukemia bodies (APBs), frequent telomere sister chromatid exchanges (T-SCEs) and recombination between telomeres from different chromosomes [58]. Telomeres in ALT cells are heterogeneous in length and relatively long, demonstrating telomere lengths much longer than telomerase-positive cells on average [22]. ALT provides cancer cells with stabilizing telomere maintenance in a telomerase-negative setting. Although it remains unclear how ALT becomes activated, its presence has been tightly associated with loss-of-function events targeting the α-thalassemia/mental retardation syndrome X-linked (*ATRX*) or death-domain-associated protein (*DAXX*) genes and these events are also a hallmark feature of IDH-mutant astrocytomas [84, 98, 104]. *ATRX* functions as an ATP-dependent helicase within the SWI/SNF family and combined these two genes form the ATRX–DAXX complex, which functions as a histone chaperone to deposit the histone variant H3.3 at telomeres [71]. Telomeric DNA has a tendency to form secondary quadruplex structures that challenge the replication machinery and need to be resolved for proper replication [170]. How exactly these two genes protect telomeres from recombination and ALT is still unknown. It has been suggested that the combined helicase activity of *ATRX* and the histone chaperone capabilities of the ATRX–DAXX complex can resolve the secondary quadruplex structure at telomeres, thereby enabling proper progression of the replication fork during S-phase and preventing the inadvertent activation of recombination (ALT) mechanisms [121, 122].

The presence or absence of inactivating *ATRX* and *DAXX* mutations present a strong correlation with ALT in many tumor types including gliomas [84, 133, 191]. Recent in vitro studies have shown that knockout of *ATRX* alone is insufficient to cause ALT; however, *ATRX* knockout combined with inactivation of p53 and RB enzymatic activity led to an increased incidence of ALT after enduring several cycles of telomere induced crisis [40, 155, 180]. Furthermore, the reintroduction of *ATRX* expression in *ATRX* mutant ALT cells led to a repression of T-SCE, APBs and c-circle formation [40, 155].

#### IDH-mutant oligodendrogliomas, 1p/19q-codeleted

Oligodendrogliomas, IDH-mutant and 1p/19q-codeleted almost universally use telomerase to maintain telomeres and virtually all of these tumors carry an activating *TERT* promoter mutation [106]. These *TERT* promoter mutations are amongst the most common non-coding mutations in cancer [89, 91, 106, 223]. Recurrent hotspot point mutations substitute a cytosine at − 228 or − 250 relative to the promoter to a thymine (C228 > T or C250 > T) to create a de novo e-twenty-six (*ETS*) transcription factor binding site that recruits the ETS family member GA-binding protein alpha chain (*GABPA*) to activate transcription [13].

Although the timing of *TERT* promoter mutations is still under debate, several lines of evidence suggest that *TERT* promoter mutations arise early in gliomagenesis and perhaps even occur prior to the glioma initiation event. *TERT* promoter mutations preferentially occur in tissues with a lower rate of self-renewal and there are numerous reports on the extra-telomeric functions of *TERT*, including effects on the NF-κB and WNT/β-catenin pathway promoting tumor growth and invasiveness [106, 141, 169]. Combined, this raises the possibility that these mutations may contribute to tumorigenesis via other pathways than its effect on telomerase alone, providing a biological reason for these mutations to contribute to gliomagenesis early in phase I and before the onset of dysfunctional telomeres. In a glioma-specific analysis, it was found that nearly all tumors with the ‘phase I event’ + 7/− 10 or ‘phase II event’ 1p/19q-codeletion have *TERT* promoter mutations, whereas not all *TERT* promoter mutant gliomas have + 7/− 10 or 1p/19q-codeletions, which may indicate that *TERT* promoter mutations even precede + 7/− 10 and 1p/19q-codeletions [34]. Another group studied the mutation fraction using multisector sequencing in 1p/19q-codeleted oligodendrogliomas and found that *TERT* promoter mutations indeed are early events and may occur before IDH mutations [204]. The idea that *TERT* promoter mutation occurs early is further corroborated by the finding that genetically engineered *TERT* promoter mutations in telomerase-positive embryonic stem cells do not affect telomerase activity, while upon differentiation these engineered cells remain telomerase positive and acquire immortality [36]. More recently, it was found that *TERT* promoter mutations in melanoma initially do not support telomere maintenance and telomeres shorten to critically short length despite harboring promoter mutations [37]. The effect on telomere length was not observed until later, which the authors attributed to a two-step immortalization process. One study even reported canonical (− 228 and − 250) somatic *TERT* promoter mutations in the blood of multiple non-cancer patients, indicating that these events could even occur before the onset of cancer and act to prime the tumor bed [143]. Although roles for *TERT* outside of telomere maintenance remain to be understood, these observations provide a sound argument that *TERT* promoter mutations can occur early in or even before gliomagenesis while providing a means towards immortalization at a later stage.

#### IDH-wildtype diffuse astrocytomas

The majority of IDH-wildtype diffuse gliomas use telomerase for telomere maintenance [120]. Re-analysis of previously published samples reclassified according to WHO 2016 criteria demonstrated that approximately 75% of diffuse IDH-wildtype gliomas are *TERT* promoter mutant [172]. Thus, *TERT* promoter mutations are common in both the most and the least aggressive diffuse gliomas (IDH-wildtype diffuse astrocytomas and IDH-mutant and 1p19q-codeleted oligodendrogliomas, respectively), suggesting that *TERT* promoter mutations are not dictating their biological behavior. It was further found that *ATRX* mutations occur in approximately 5% of IDH-wildtype diffuse astrocytomas [172]. The prevalence of ALT in IDH-wildtype diffuse gliomas is higher than the frequency of *ATRX* mutations, suggesting that some of these tumors may use ATRX-independent mechanisms to activate ALT [85]. In a similar fashion, the prevalence of telomerase activity is higher than the prevalence of *TERT* promoter mutations in this tumor type, suggesting that these tumors may use *TERT* promoter-independent mechanisms for the reactivation of telomerase [120]. Several candidate mechanisms have been previously described in glioma, including *TERT* promoter methylation or *TERT* amplifications [11]. Contrary to adult IDH-wildtype diffuse glioma, H3-mutant malignant pediatric glioma frequently demonstrates ALT [140], and several studies reported frequent co-occurrence of *ATRX* mutations in both H3 K27 and G34 mutant gliomas. However, reports of co-occurrence vary between 30 and 60% for K27 and 75–100% for G34, suggesting that there is a role for telomerase in many of these tumors as well [123].

### Phase V: immortalization and dedifferentiation

The glioma stem cell theory states that amongst all cancerous cells in a tumor, a subset of cells act as progenitor or stem cells with reproductive capabilities and sustaining the cancer, much like normal bone marrow stem cells are responsible for replenishing the population of circulating leukocytes [221]. It has often been contrasted to the theory of clonal evolution, which suggests that cancers evolve through an iterative process of clonal expansion from a single cell [73]. Recent advances in single-cell sequencing and lineage tracing have unveiled multiple populations of tumor cells in bulk tumor samples, providing fuel for the cancer stem cell hypothesis [119, 171, 210, 219]. One study used single-cell RNA sequencing on IDH-mutant and 1p/19q-codeleted oligodendroglioma patient samples and uncovered distinct cell populations of undifferentiated tumor stem cells and cells that are more differentiated along various glial lineages [210]. In a similar study of IDH-mutant astrocytoma the authors were able to detect the same cellular populations but with a higher ratio of stem-like to differentiated cells that increased with increasing WHO grades [219]. Another study used DNA barcoding of repeatedly in vivo-transplanted glioblastoma cells to trace the lineage during their engraftment and found a population of progenitor cells that sustained the tumor and gave rise to differentiated non-proliferative cells [119].

**Fig. 4:**
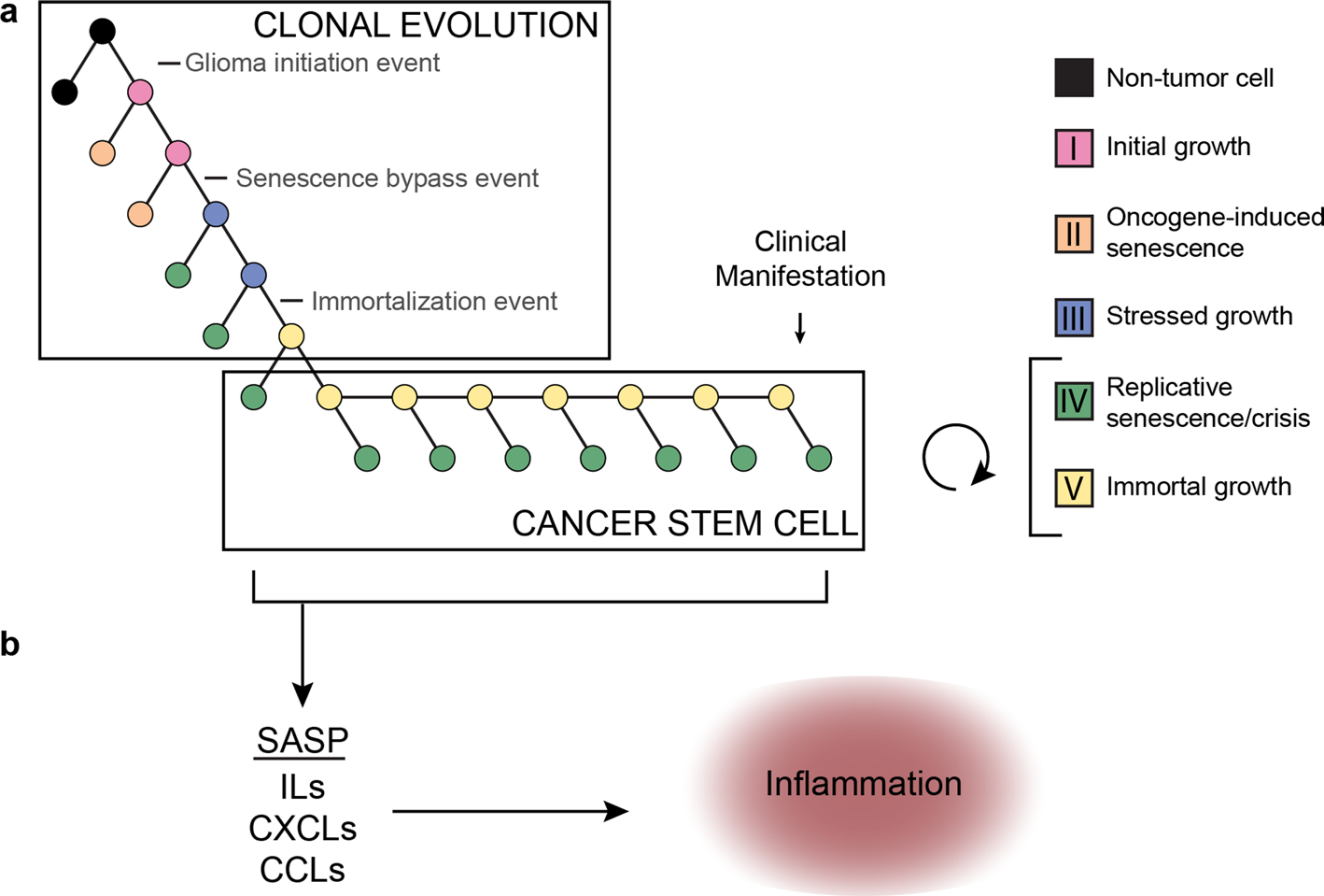
**a** Integration of a clonal evolution and cancer stem cell model for gliomagenesis. This model assumes that sequential mutations and selection pressure drive the evolution of cancer stem-like cells. At the same time, these stem-like cells may give rise to more differentiated (i.e., phase IV) offspring that may divide further but rapidly become growth arrested. **b** According to this model these cells may be senescent and contribute to the cancer phenotype by eliciting a microenvironment response via SASP. *SASP* senescence-associated secretory phenotype; *ILs* interleukins; *CXCLs* chemokines (C–X–C motif); *CCLs* chemokines (C–C motif)

These studies all provide support for a cancer stem cell hypothesis and raise the question how these findings fit with our model, which leans towards a model of clonal evolution. In fact, current evidence may suggest that both mechanisms are acting together (Fig. 4a). Whereas clonal evolution is important to establish the initial cancer stem cell population, neutral evolution (in line with the cancer-stem cell hypothesis) may fit better once the initial cancer core has been established, especially so when further evolutionary stimuli (e.g., senescence barriers, hypoxia, treatment) are lacking. The concept of neutral evolution holds that most molecular changes are not caused by natural selection but rather by the stochastic allelic variation that are neutral and do not affect cellular fitness [109]. A recent study analyzed cancer genomes from TCGA and found neutral evolution in approximately one-third of a wide spectrum of over 900 tumors, including 35 gliomas of which 8 (23%) suggested evidence in support of a neutral evolution process [236]. The authors conclude that all clonal selection must have occurred before the onset of cancer growth and not in later arising subclones. Several groups have since challenged these findings and suggested that their analysis does not univocally prove neutral evolution starting from the first malignant cell [157, 207, 237]. While it may be possible that tumors evolve neutrally beyond the most recent selective sweep we do not agree with the conclusion that these findings suggest that all clonal selection must occur before the onset of tumor growth. The authors do not take into account that at the time the tumor presents itself and is surgically removed, all remnants of a selection process that have been outcompeted or died will have completely disappeared, in contrast to “neutral” variants which do not affect fitness and will remain. According to our simplified model, tumorigenesis sequentially follows phase I–V. Once the cancer stem cell population has been established, tumor cells will follow neutral evolution, as long as new evolutionary or other stimuli for opportunistic growth are lacking (Fig. 4a).

Aforementioned studies supporting the cancer stem cell hypothesis suggest that multiple cellular populations exist within a tumor, including a self-renewing cancer stem cell population and a less-proliferative differentiated population. A recent paper demonstrated that cells derived from glioma stem cells may differentiate and subsequently undergo senescence [167]. We speculate that phase V tumor cells represent cancer stem cells that may give rise to more differentiated, phase IV cells (Fig. 4a). The transition from phase IV to V and vice versa is likely volatile in nature owing to transcriptional reprogramming including the activation of the stemness factors oligodendrocyte transcription factor 2 (*OLIG2*), sex-determining region Y-box 2 (*SOX2)* and the reactivation of telomerase [203]. On the other hand, the transition from phase I to phase IV is more rigid in nature, involving various changes on a genomic level, including somatic mutations and copy number changes as described. This important distinction in flexibility led us to believe that phase V cells may re-differentiate and assume a phase IV state.

Although they are growth arrested, senescent cells are metabolically active and release a plethora of signaling molecules to the microenvironment. The senescence-associated secretory phenotype (SASP) is a feature of senescent cells that curtails these cells with the release of proinflammatory cytokines [47]. SASP does not depend on p16^INK4a^ or p21 activity and senescence with intact p16^INKa^ function actually suppresses SASP [49]. Similarly, activated p53 signaling also suppresses SASP while *TP53* loss induces SASP [48, 136, 178]. These findings suggest that the SASP response is stronger when senescence pathway genes are lost. Indeed, IDH-wildtype astrocytomas often harbor homozygous deletions in *CDKN2A/B* and are known to have a highly active microenvironment [230]. Genes associated with SASP were shown to be overexpressed in higher grades of glioma and older patients, the latter group more likely to be affected by high-grade IDH-wildtype astrocytoma [50]. Moreover, it was found that primary glioblastoma cells retain a functional senescence program despite mutations in the *TERT* promoter and *CDKN2A/B* locus [118]. These findings imply that senescent and differentiated phase IV cells may be crucial for shaping the immune microenvironment in gliomas (Fig. 4b).

## A broader perspective

While there are not many known environmental risk factors that predispose to glioma, large cohort genome-wide association studies over the past two decades have identified multiple heritable polymorphisms conferring glioma risk [96, 110, 152, 179, 196, 238]. Notably, several of these risk loci are localized to genes involved in telomere maintenance, including the telomerase reverse transcriptase *TERT*, telomerase RNA component *TERC*, and other telomere maintenance-associated genes STN1, CST complex subunit (*OBFC1)*, protection of telomeres 1 (*POT1*) and regulator of telomere elongation 1 (*RTEL1*) [152]. Moreover, there appears to be a significant increased glioma risk in people with increased leukocyte telomere lengths [45]. Unsurprisingly, glioma risk alleles at aforementioned genes are also associated with increased leukocyte telomere length [41, 226, 228]. Telomeres thus play an important role in not only the development of gliomas, but also in glioma risk [227]. In fact, the positive association between leukocyte telomere length and cancer risk is not specific to glioma and shared across many cancers. A recent Mendelian randomization study found that longer leukocyte telomere length was associated with an increased risk to cancer but a reduced risk to non-neoplastic disease such as cardiovascular disease [209]. It has been suggested that this difference is due to individuals with longer telomeres being more likely to acquire driver mutations due to an increased proliferative potential whereas the inverse relationship observed for non-neoplastic disease may be due to the impact of telomere shortening on tissue degeneration [19, 200].

Several hereditary disorders are associated with an increased risk of glioma development, including neurofibromatosis type 1 and type 2 (NF1, NF2) and the *TP53* germline mutation/Li–Fraumeni syndrome. NF1 and NF2 are autosomal dominant hereditary disorders with germline mutations in *NF1* and *NF2* and clinically characterized by multiple benign nerve sheath tumors (especially neurofibromas in NF1, schwannomas in NF2), but also by a markedly increased risk on particular gliomas (especially pilocytic astrocytoma in NF1 and ependymomas in NF2) [33, 76, 184]. Both genes are well-known tumor suppressor genes and key components in the MAPK pathway [38]. It has been demonstrated that senescence commonly occurs in benign nerve sheath tumors and that prolonged *NF1* disruption leads to oncogene-induced senescence in a model system, providing a rationale as to why these germline disorders present with tumors that are often relatively indolent [52]. A germline perturbation affecting *NF1* or *NF2* can be considered a tumor initiation event, explaining why this germline disorder guarantees the formation of multiple benign nerve sheath tumors over one’s lifetime.

Li–Fraumeni syndrome is a rare autosomal dominant hereditary disorder that is caused by the germline perturbation of *TP53* or *CHK2*, which regulates p53 activity [12, 18, 146]. Whereas NF1 and NF2 guarantee the formation of especially multiple benign tumors (including non-diffuse gliomas) in a lifetime, Li–Fraumeni patients pose a greater risk to developing a malignant tumor, including a diffuse glioma [17]. This risk increases with age and is over 50% at age 30, with a lifetime cancer risk of over 70% in men and almost 100% in women [146]. Moreover, 15 and 4% of affected individuals were found to develop a second and third cancer [86]. Li–Fraumeni syndrome germline mutations affect phase II and prevent the onset of oncogene-induced senescence following the acquisition of a glioma initiation event, thus increasing the risk of developing cancer over a lifetime.

The germline mutations underlying NF1/NF2 and Li–Fraumeni syndrome represent pathways that both need to be disrupted for a malignant tumor to form. The fact that nearly all patients with NF1/NF2 develop one or more benign tumors can be understood by acknowledging that in these disorders a germline tumor initiation event is involved. Unless this pathway is supplemented by a senescence bypass event, these tumors do not readily proceed to malignancy. In contrast, Li–Fraumeni syndrome is characterized by an increased risk for malignant tumors in many (but not all) patients. In this syndrome, the germline senescence bypass event needs to be supplemented by a tumor initiation event for a tumor to develop, and such tumors may be more aggressive/malignant due to the defective senescence barrier, allowing the tumor (precursor) cell to instantly progress to phase III and instigate genomic instability.

## Conclusion

Our knowledge on the molecular events driving cancer has grown exponentially over the years. This review has aimed to put this new knowledge into the perspective of the temporal molecular pathogenesis of glioma, starting from the first aberrant cell all the way to a symptom-causing glioma. To this end we have combined what is known on gene (mal)function, tumor evolution, genomic instability and telomere maintenance to develop a model of gliomagenesis. This model describes five sequential phases cancer cells undergo during their gliomagenesis. We speculate that transitions from one phase to the next can be characterized by acquisition of tumor-driving events that sequentially contribute to the hallmarks of cancer as previously proposed, including proliferation, evasion of apoptosis and limitless replicative potential [79, 80]. Our model is a simplified abstraction of what may be the truth and new insights will refine and improve our understanding. Meanwhile, we hope that our model will help foster hypotheses leading to new insights into the molecular life history of glioma that will help identify convincing therapeutic vulnerabilities.
